# Percutaneous thrombectomy assisted lead extraction: successful diagnosis and treatment in a case report of culture negative endocarditis

**DOI:** 10.1093/ehjcr/ytaf086

**Published:** 2025-02-18

**Authors:** Sujoy Khasnavis, Jeffrey Green, Jacob Cynamon, Jay Gross

**Affiliations:** Montefiore Medical Center Moses Campus, Albert Einstein College of Medicine, 111 East 210th St, Bronx, NY 10467, USA; Montefiore Medical Center Moses Campus, Albert Einstein College of Medicine, 111 East 210th St, Bronx, NY 10467, USA; Montefiore Medical Center Moses Campus, Albert Einstein College of Medicine, 111 East 210th St, Bronx, NY 10467, USA; Montefiore Medical Center Moses Campus, Albert Einstein College of Medicine, 111 East 210th St, Bronx, NY 10467, USA

**Keywords:** Case report, *P. acnes*, AngioVac extraction, ICD endocarditis, ICD extraction, Native tricuspid valve endocarditis, Atypical pathogen

## Abstract

**Background:**

*Propionibacterium acnes* is a rare cause of prosthetic valve endocarditis and implantable cardiac device [implantable cardioverter defibrillator (ICD)] endocarditis. Previous reports of *P. acnes* endocarditis have described various approaches to treatment. Treatment of *P. acnes* endocarditis with AngioVac extraction (AE) is unreported. We describe a case of *P. acnes* ICD endocarditis that required AE to facilitate device extraction and ensure successful treatment of endocarditis.

**Case summary:**

The patient presented with classical symptoms of fever, fatigue, night sweats, and chills for 3 weeks. Serial blood cultures were negative. Cultures from skin were positive for *P. acnes*. Transthoracic endocardiogram and transoesophageal echocardiogram demonstrated vegetations adjacent to the transvenous leads and on the septal leaflets of the native tricuspid valve (NTV). Due to vegetation size, AE was performed to debulk the vegetations prior to lead extraction (LE). Vegetation cultures were also positive for *P. acnes*. He was prescribed i.v. antibiotics post-operatively and had an excellent long-term course with no further endocarditis episodes or complications at 3-year follow-up.

**Discussion:**

Our case is one of the first to demonstrate the extent to which the integumentary pathogen *P. acnes* is involved in cardiac infections. It is also the first literature-reported case to utilize AE to treat *P. acnes* ICD endocarditis involving the NTV. This case illustrates the utility of AE in vegetation debulking prior to ICD LE and in identifying atypical pathogens where conventional diagnostic tests are unable to detect a pathogen.

**Learning points**

- AngioVac aspiration of cardiac vegetations can diagnose rare causes of endocarditis such as *Propionibacterium acnes* when conventional testing cannot reliably or readily identify causative pathogens.
- AngioVac-guided vegetation debulking can facilitate the extraction of infected cardiac device leads and the subsequent treatment of endocarditis by antibiotics.

## Introduction

*Propionibacterium acnes* (*P. acnes*) is an anaerobic component of normal human skin flora and considered a minor contributor to systemic infections.^1,2^ It is a very rare cause of infectious endocarditis and is more commonly seen in prosthetic valve endocarditis and implantable cardioverter defibrillator (ICD) endocarditis than in native structure infections including native tricuspid valve.^2^  *Propionibacterium acnes* endocarditis commonly affects men with prosthetic valves, and findings of *P. acnes* in blood cultures favour this diagnosis. The challenge of diagnosing *P. acnes* endocarditis remains in the fact that blood cultures are frequently negative and when positive are considered culture contaminants.^3^ Diagnosis, therefore, requires other testing methods such as incubation of blood cultures over two weeks time or sequencing of pathogens from infected valves, the latter having high success.^3^ However, in cases where treatment is time sensitive and routine test options are not available, the AngioVac system has been utilized to debulk valvular and device vegetations and facilitate the treatment of endocarditis.^4–7^ In this report, we elaborate on the case of a 63-year-old male who presented with cardiac device infection and required AngioVac extraction (AE) for identification and treatment of *P. acnes* endocarditis.

## Summary figure

Endocarditis diagnosis and treatment with pre-, peri-, and post-procedure images.

**Figure:**
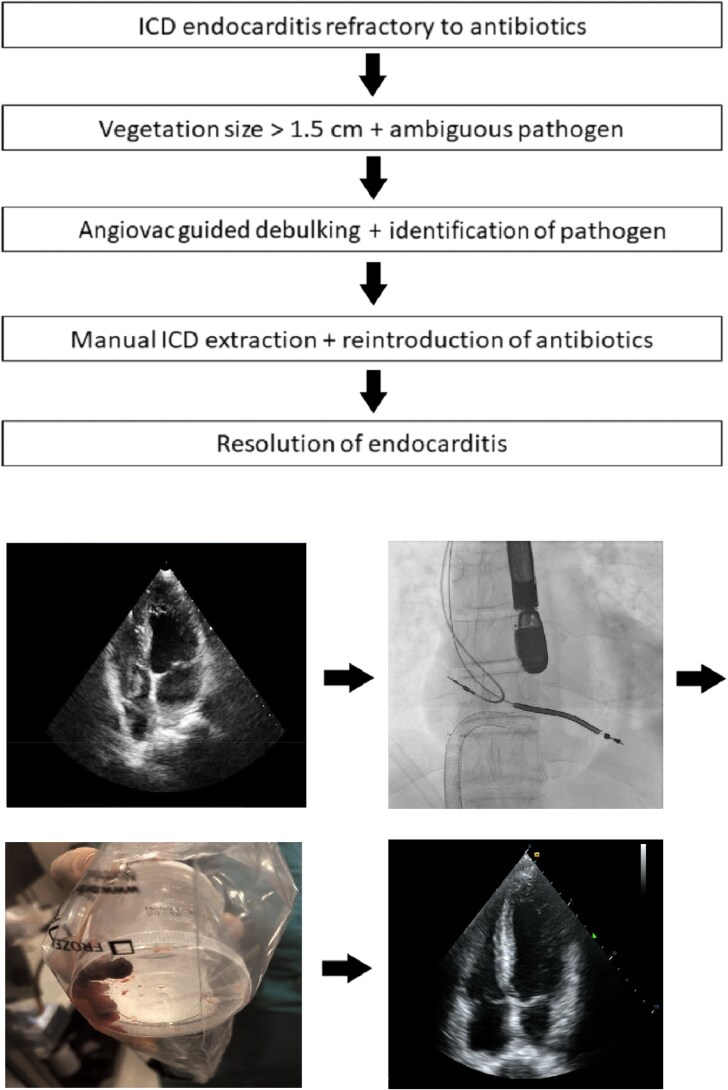


## Patient information

The patient was a 63-year-old white non-Hispanic, non-Latinx male with history of hypertension (HTN), coronary artery disease of the left anterior descending (LAD) artery, ST elevation myocardial infarction (STEMI) s/p coronary artery bypass graft from left internal mammary artery to LAD artery and percutaneous coronary intervention, and cardiac arrest s/p implantation of a transvenous dual-chamber Medtronic ICD [right atrium (RA)—5076, right ventricle (RV)—6935M single coil]. The ICD was implanted three years prior to presentation. He presented to a community hospital with fever, chills, cough, fatigue, and night sweats for 3 weeks. He denied sick contacts, travels to endemic locations, exposures to high risk populations, intravenous (i.v.) drug use, alcohol use, smoking, medications, and allergies. He was a retired music instructor living with his spouse who had no communicable illnesses. He had family history of HTN.

Vitals were 36.7°C, heart rate 62 b.p.m., respirations 18 b.p.m., blood pressure 129/81 mmHg, and SpO2 100% on room air. The skin of the face, neck, and back was notable for comedones. Cardiac examination was notable for a mild systolic murmur along the left parasternal border at the third intercostal space. The pulse generator was seated in a subcutaneous pocket at the upper left pectoral area and well-healed without erythema, tenderness, swelling, or compromise in the skin. The remainder of the examination was unremarkable.

Laboratory testing showed an elevated white count of 14.3 k/μL (ref. 4.8–10.8 k/μL), low haemoglobin of 12.4 g/dL (ref. 14.0–17.4 g/dL), and low haematocrit of 39.3% (ref. 41.5%–50.4%). All other labs were within normal limits including sodium 136 mEq/L (ref. 135–145 mEq/L), potassium 4.4 mEq/L (ref. 3.5–5.0 mEq/L), chloride 102 mEq/L (ref. 98–108 mEq/L), bicarbonate 27 mEq/L (ref. 20–30 mEq/L), urea nitrogen 12 mg/dL (ref. 5–20 mg/dL), creatinine 0.72 mg/dL (ref. <1.30 mg/dL), glucose 96 mg/dL (ref. 70–140 mg/dL), calcium 8.6 mg/dL (ref. 8.5–10.5 mg/dL), anion gap 7 mEq/L (ref. 7–16 mEq/L), magnesium 2.1 mg/dL (ref. 1.7–2.8 mg/dL), phosphate 2.5 mg/dL (ref. 2.5–4.5 mg/dL), albumin 3.6 g/dL (ref. 3.5–5.0 g/dL), bilirubin 0.5 mg/dL (ref. <1.2 mg/dL), alkaline phosphatase 73 U/L (ref. <130 U/L), alanine aminotransferase 23 U/L (ref. <40 U/L), aspartate aminotransferase 18 U/L (ref. <50 U/L), protein 6.1 g/dL (ref. 6.0–8.0 g/dL), prothrombin time 13.8 s (ref. 11.8–14.8 s), and partial thromboplastin time 33.8 s (ref. 25.9–38.9 s).

Electrocardiogram on admission showed normal sinus rhythm with borderline left ventricular hypertrophy. Ventricular rate was 71 b.p.m., PR interval 164 ms, QRS duration 90 ms, QT 398 ms, and QTc 432 ms.

Aerobic and anaerobic cultures of the facial acne were positive for *P. acnes*. Aerobic and anaerobic blood cultures were negative for organisms after multiple tests. On chest X-ray, lungs showed small bilateral pleural effusions only. The chest CT was remarkable for multifocal pneumonia with cavitations suggestive of septic emboli.

**Figure 1:**
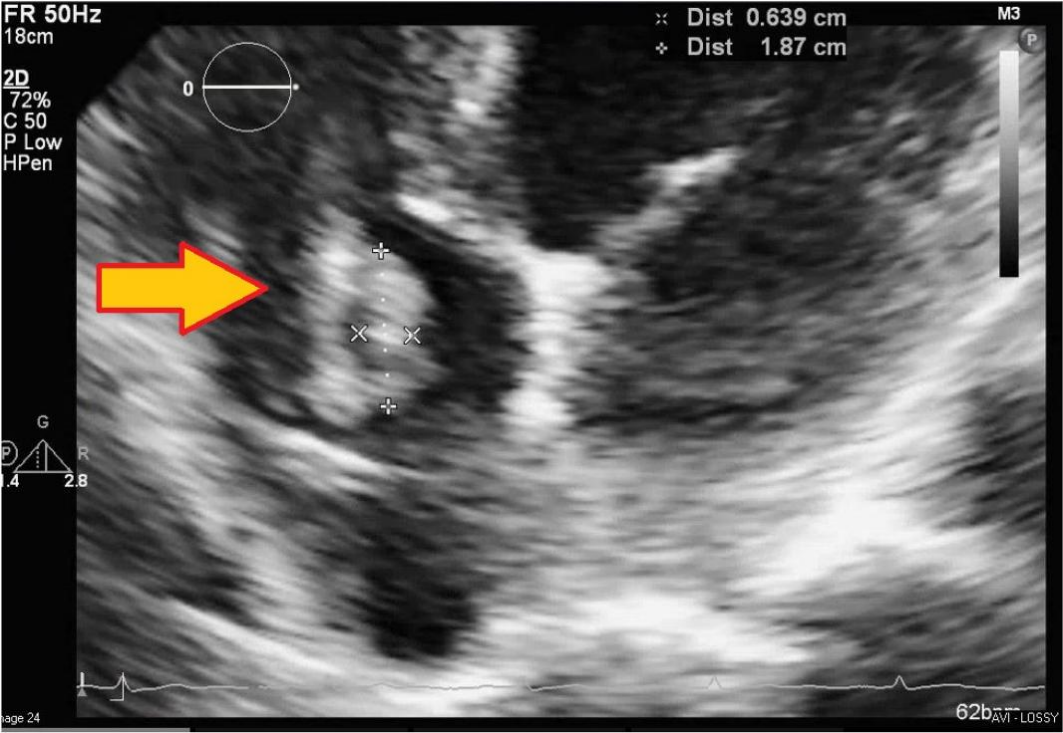
Transthoracic echocardiogram with RA mass of dimensions 2 cm × 3 cm near tricuspid valve (arrow).

No official echocardiogram recordings or video clips were made before, during, or after the AE procedure, and live recordings were, therefore, unavailable. Transthoracic echocardiogram (TTE) (*Figure 1*) showed a RA of normal size and function with a pacemaker lead containing a 2 cm × 3 cm mass on a pacing wire near the tricuspid valve. Left ventricle function and size were normal with an ejection fraction of 60%. The tricuspid valve had a mass close to the septal leaflet, prolapsing through the valve and inducing mild regurgitation. The remaining chambers and valves were grossly normal in size, structure, and function. No pericardial effusion was noted.

The patient’s presentation was initially thought to be a manifestation of community acquired pneumonia. He was thereby initiated doxycycline therapy. When doxycycline failed to adequately treat symptoms, he was transitioned to levofloxacin. Levofloxacin also did not produce a substantial response. After TTE showed ICD vegetations, he was started on vancomycin and ceftriaxone. However, no adequate response was obtained from any of these regimens. Due to vegetation size, ambiguity of the offending pathogen, and failure to achieve response to antibiotics, he was transferred to our institution for further management.

**Figure 2:**
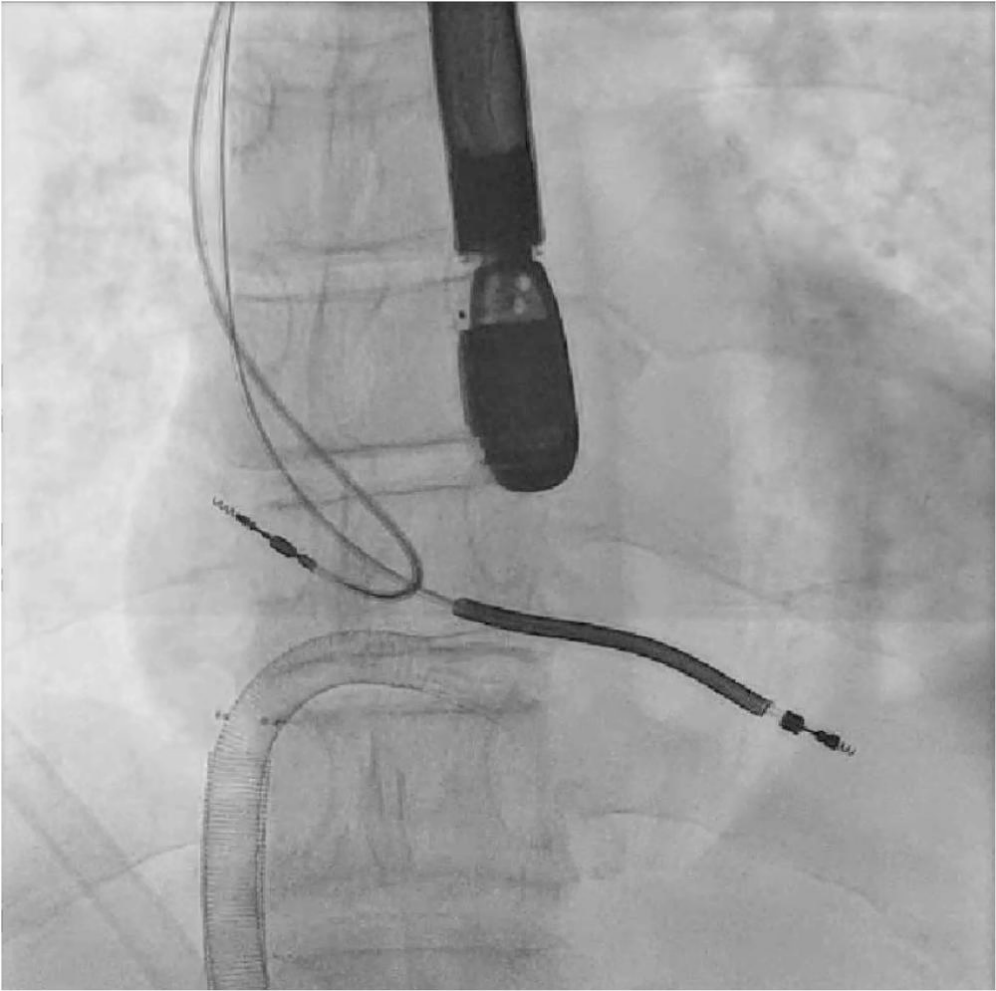
Intraoperative fluoroscopy of AngioVac catheter and leads (pre-extraction).

**Figure 3:**
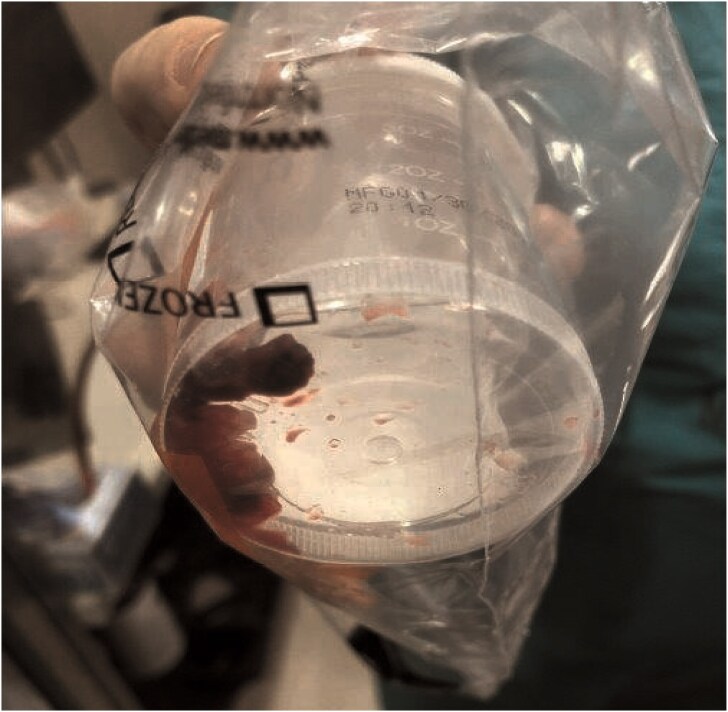
Collection of endocardial vegetation fragments acquired from AngioVac aspiration.

At our hospital, the decision was made to extract the ICD. Although no Heart Team meeting was held, the electrophysiology team came to the conclusion that extraction was necessary since prior therapies had failed. AngioVac debulking was performed with interventional radiology guidance. Access was acquired with a right femoral 26 Fr catheter and a left femoral 16 Fr catheter. An AngioVac Gen3 180 catheter was advanced from the right femoral catheter to the RA under fluoroscopy (*Figure 2*). Transoesophageal echocardiogram (TEE) guidance was used to position the AngioVac catheter proximate to the vegetations and begin debulking ICD lead and tricuspid annulus vegetations. Multiple large fragments were removed using this approach (*Figure 3*). Intraoperative TEE confirmed the absence of myocardial and valvular injury with a small residual vegetation remaining adherent to the tricuspid valve. No further attempt was made at debulking due to the concern about tricuspid injury. The vegetations were sent to the microbiology lab. The device pocket was opened, and cultures of the pocket were sent. Right atrium and RV leads were transected and removed by direct manual traction alone. Utilizing sharp and blunt dissection, the pulse generator was removed from the pocket and detached from the leads.

**Figure 4:**
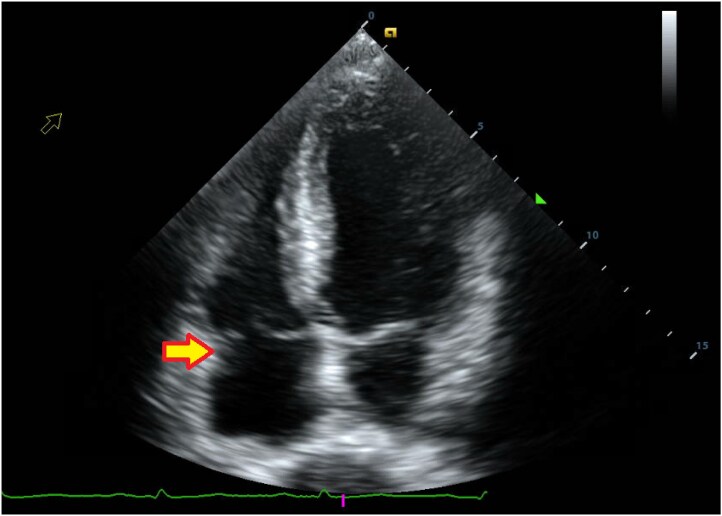
Post-procedural apical four-chamber TTE showing RA after extraction of vegetations and masses. Very small residual vegetation along the RA (arrow).

Transthoracic echocardiogram after procedure showed a 0.5 cm residual vegetation attached to chordae and septal tricuspid leaflet (*Figure 4*). Implantable cardioverter defibrillator leads were no longer present, and the remainder of the findings were same as pre-procedure.

Following AE of the ICD system, the patient had no acute complications. Six days after procedure, aerobic and anaerobic cultures of the vegetation fragments grew *P. acnes*. Device pocket cultures did not grow any organisms. Infectious disease was consulted and recommended transitioning vancomycin to daptomycin. On discharge, antibiotics were adjusted and he was sent home on four weeks of i.v. daptomycin and ceftriaxone. One month later, he was doing well and had no symptoms or signs of reinfection. Three years later at cardiology clinic, he was free of symptoms and had no recurrence of endocarditis or other cardiac events.

The benefits vs. risks of ICD reimplantation including subcutaneous implantable cardioverter defibrillator reimplantation were weighed, and the decision was made to avoid reimplantation. The reasons for this decision were three-fold. First, TTE showed a normal range ejection fraction. Second, the original ICD had been implanted after one instance of VT due to STEMI from which the patient had long since recovered. Third, the involvement of a cutaneous pathogen in endocarditis presented a significantly elevated risk for reinfection and thus it was prudent to avoid reimplantation.

## Discussion

*Propionibacterium acnes* has historically been regarded as a cutaneous pathogen that is uncommon in endocardial infections.^3^ This is in part due to the frequency of staphylococcal, streptococcal, and enterococcal infections as compared to *P. acnes*. It is also due to the notion that *P. acnes* is a blood contaminant even if it is identified in serum cultures.^1^ Multi-centre studies have further reported that *P. acnes* accounted for 14/2500 endocarditis cases of which 12 occurred with prosthetic devices.^8^ Others have reported on the challenges of detecting *P. acnes* endocarditis by routine methods and the utility of both polymerase chain reaction and extended incubation periods for blood cultures.^3,12–15^ Collectively, the literature demonstrates that *P. acnes* cardiac infections are difficult to diagnose unless fulminant or through highly specialized tests.

Prior to the development and use of percutaneous thrombectomy devices, percutaneous lead extractions were often performed on large clots in major circulatory vessels regardless of clot size.^9^ At our institution, extractions have been performed on intravascular masses up to 2.5 cm. However, these practices are associated with a considerable risk of pulmonary embolism and persistence of bacteraemia. This case is a prime example where AE provided an endocardial sample for identifying and treating *P. acnes* with minimal complications. AngioVac debulking ensured that lead extraction (LE) would present a very low risk of embolism to the pulmonary circulation and that subsequent antibiotic therapy would eradicate the infection. The whole ICD system was extracted in this process. AngioVac extraction ultimately led to a microbial diagnosis that was conventionally unobtainable and thereby aided in rapid recovery. This highlights the benefits of a vegetation debulking strategy prior to LE. In other literature-reported cases, the use of the Inari and Indigo systems has been well documented.^10,11^ These systems, however, were not familiar to our team and, therefore, not utilized in our case.

## Conclusion

*Propionibacterium acnes* is a rare cause of endocarditis that is challenging to diagnose through conventional approaches. The AE system is highly effective and accurate in catching *P. acnes* and other rare endocarditis pathogens when conventional tests are inconclusive. The system is also therapeutically useful as it debulks ICD vegetations and facilitates both LE and antibiotic treatment with low risk for complications.

## Data Availability

All data are incorporated into the article.

## Lead author biography

Dr Sujoy Khasnavis is a PGY-3 Internal Medicine Resident with Montefiore and Jacobi North Central Bronx Hospitals. He is a professional member of ESC and recent clinical case presenter at ESC Congress 2024.

## Notes

**Consent:** In line with COPE guidelines, informed consent was obtained from all identifiable individuals along with their right to withdraw consent. Participants understood the potential implications of their information being published, and they freely agreed to publication. Appropriate measures were taken to anonymize details where necessary.

**Funding:** No funding was obtained or required for this publication.
